# Analysis of risk factors for delayed intracranial hemorrhage following ventriculoperitoneal shunt surgery and construction of a nomogram model

**DOI:** 10.3389/fneur.2025.1721488

**Published:** 2025-12-18

**Authors:** Guan-Jiang Lin, Hong-Cai Wang, Shi-Wei Li

**Affiliations:** The Affiliated Lihuili Hospital of Ningbo University, Ningbo, Zhejiang, China

**Keywords:** delayed intracranial hemorrhage, hydrocephalus, influencing factor, nomogram, ventriculoperitoneal shunt

## Abstract

**Objective:**

The objective of this study is to identify the independent risk factors associated with delayed intracranial hemorrhage (DICH) subsequent to ventriculoperitoneal shunt (VPS) surgery in patients diagnosed with hydrocephalus and to construct a nomogram model for this condition.

**Methods:**

We conducted a retrospective analysis of clinical data from 266 patients who underwent VPS procedures at Ningbo University Affiliated Lihuili Hospital between January 2015 and December 2024. The patients were stratified into two groups: those with postoperative DICH (DICH group) and those without (non-DICH group). Initially, univariate analysis was used to evaluate differences in clinical data between the two groups. Subsequently, multivariate logistic regression analysis was employed to identify independent risk factors for DICH (*p* < 0.05); R software was used to construct a nomogram model for predicting the occurrence of delayed intracranial hemorrhage (DICH) following ventriculoperitoneal shunt (VPS) surgery. Finally, the receiver operating characteristic (ROC) curve and calibration curve were employed to comprehensively evaluate the predictive performance of the nomogram model.

**Results:**

The incidence of DICH was observed to be 17.3% (46/266). Statistical analysis revealed that the DICH group had significantly higher age, a greater prevalence of previous craniotomy, and an elevated postoperative-to-preoperative neutrophil-to-lymphocyte ratio ratio (NLRR) compared to the non-DICH group (*p* < 0.05). Multivariate logistic regression analysis identified age (OR = 1.061, 95% CI: 1.021–1.103), a history of craniotomy (OR = 2.676, 95% CI: 1.196–5.989), and NLRR (OR = 1.931, 95% CI: 1.373–2.717) as independent risk factors for DICH (*p* < 0.05). The results of ROC curve analysis showed that the Area Under the Curve (AUC) was 0.80 (95% CI: 0.73–0.87). The sensitivity was calculated to be 87.7%, the specificity was 60.9%, the positive predictive value (PPV) was 91.5%, the negative predictive value (NPV) was 50.9%, and the cutoff value was 0.254. Additionally, the Hosmer-Lemeshow (HL) test for goodness-of-fit yielded a chi-square value (*χ*^2^) of 10.145 and a *p*-value of 0.255, indicating that the model has good calibration.

**Conclusion:**

Advanced age, a history of craniotomy, and elevated NLRR are independent risk factors for DICH following VPS in hydrocephalus patients. The constructed nomogram model for predicting DICH occurrence after VPS surgery in hydrocephalus patients exhibits favorable predictive performance. Postoperatively, enhanced vigilance for DICH is recommend when any of these risk factors are present postoperatively. Increasing the frequency of postoperative head CT scans may improve detection sensitivity for asymptomatic patients with minor bleeding, thereby facilitating early intervention.

## 1 Introduction

Ventriculoperitoneal shunt (VPS) is a common surgical procedure for treating hydrocephalus. Nevertheless, it can be associated with a spectrum of complications, including infection, shunt obstruction, seizures, and intracranial hemorrhage, with overall incidence rates reported to range from 23.8% to 35% (1, 2).

Delayed intracranial hemorrhage (DICH) is defined as hemorrhagic events not detected on the initial cranial CT within the first 24 h after VPS surgery, but subsequently identified on follow-up imaging more than 24 h postoperatively, indicating new bleeding in the ventricles or along the catheter tract. The reported incidence of DICH varies from 0.3% to 23.7% (3–8).

The pathophysiological mechanisms underlying DICH following VPS remain adequately elucidated. Existing literature proposes several contributory factors, including: (1) increased fragility of cerebral microvasculature; (2) inflammatory responses; (3) postoperative alterations in shunt valve pressure; (4) use of antiplatelet agents (such as aspirin and clopidogrel), anticoagulants (such as low molecular weight heparin and warfarin), or the presence of coagulation disorders; and (5) peri-catheter cerebral edema.

This study undertakes a retrospective analysis the clinical characteristics of DICH following VPS in hydrocephalus patients, and employs statistical methods to identify relevant risk factors. The findings hold substantial significance in advancing the comprehension of DICH, summarizing clinical experiences, and developing preventive strategies, thereby providing valuable insights for the management of this complication.

## 2 Materials and methods

### 2.1 Clinical data

**Figure 1:**
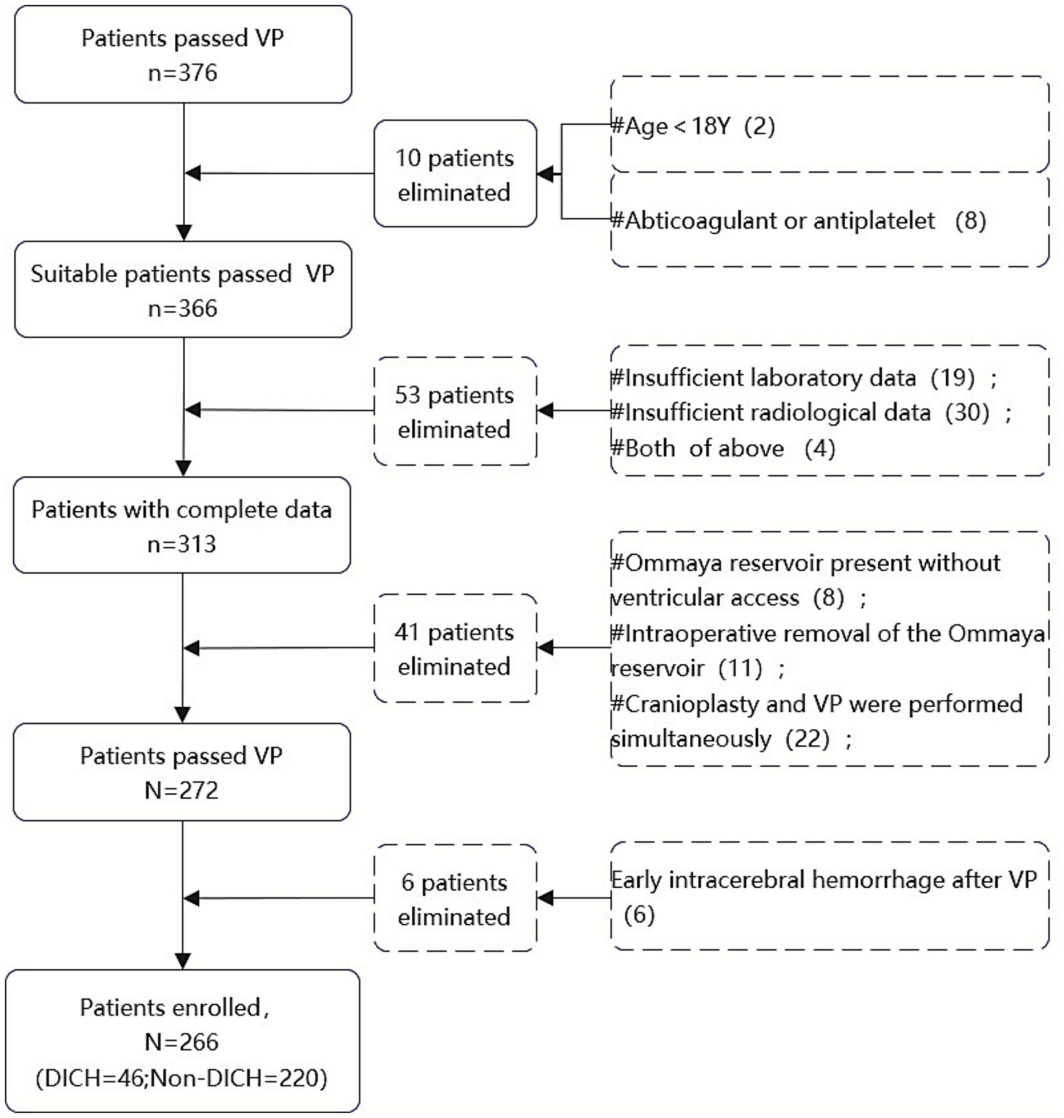
Flowchart of patient selection.

A retrospective analysis was conducted on data from 266 patients who underwent VPS surgery in the Department of Neurosurgery at Ningbo University Affiliated Lihuili Hospital between January 2015 and December 2024. All surgeries were performed by experienced senior neurosurgeons after obtaining informed consent, and complete clinical data were available. The inclusion criteria were as follows: (1) patients aged 18 years or older; (2) Patients had laboratory tests (including complete blood count, coagulation function) within 5 days before VPS and an additional complete blood count on the first morning after VPS; (3)A cranial CT scan was performed on the first day after VPS (later than the postoperative test of blood routine), and at least one additional cranial CT scan was conducted within 3–10 days postoperatively; (4) utilization of a Medtronic adjustable pressure shunt valve. Exclusion criteria included: (1) perioperative use of antiplatelet or anticoagulant medications, or pre-existing coagulation disorders; (2) early postoperative acute intracranial hemorrhage; (3) patients with Ommaya reservoir implantation, the Ommaya tube was directly connected to the shunt pump without ventricular puncture, or the Ommaya tube was removed during the surgery; (4) concurrent cranioplasty and VPS surgery; (5) use of other brands of adjustable pressure VP shunts. The patient flowchart was summarized in Figure 1.

This study was approved by the Ethics Committee of Ningbo University Affiliated Lihuili Hospital (Approval No. KY2025SL173-01). Informed consent was waived due to the retrospective nature of the analysis.

### 2.2 Definition of DICH and grouping

DICH was defined as the presence of new hemorrhage within the ventricles or along the catheter tract on follow-up CT scans performed more than 24 h postoperatively. These CT scans were performed in two scenarios: (1) Symptom-driven: performed immediately when patients presented with clinical deterioration (e.g., vomiting, seizures, decreased consciousness, or new neurological deficits); (2) Routine follow-up: performed on postoperative day 3–10 for asymptomatic patients. All follow-up CT scans revealed hemorrhage that was not present on the initial CT scan conducted within the first 24 h following VPS.

Patients were categorized into two groups based on the presence of DICH: the non-DICH group (Group A) and the DICH group (Group B). Furthermore, the Group B was subdivided into two subgroups: those who received surgical intervention (Subgroup B_1_) and those who underwent conservative management (Subgroup B_2_).

### 2.3 Management and prognostic evaluation of DICH

In the DICH group, patients underwent surgical treatment if they presented with symptoms such as vomiting, seizures, persistent decreased consciousness, and a hemorrhage volume ≥ 30 mL as determined by follow-up CT imaging; otherwise, conservative treatment was adopted. The surgical methods involved using the original incision and burr hole to create a small bone window for neuroendoscopic hematoma evacuation. In cases where the hematoma had not ruptured into the ventricle, the shunt tube remained unadjusted. Conversely, if ventricular rupture occurred, the ventricular hematoma was evacuated, and the shunt tip was cleaned and reinserted. Conservative treatment involved initially increasing the pressure of the shunt pump, followed by the administration of hemostatic, antiepileptic, and dehydrating therapies. The shunt pressure was subsequently reduced once the hematoma had stabilized.

Patient outcomes at discharge were assessed using the Glasgow Outcome Scale (GOS), where a score of 5 indicated good recovery, 4 indicated moderate disability, 3 indicated severe disability, 2 indicated a vegetative state, and 1 indicated death. A GOS score greater than 3 was classified as a favorable outcome, whereas a score of 3 or less was considered an unfavorable outcome.

### 2.4 Surgical procedure

All patients received a Medtronic adjustable pressure shunt system, which was performed by senior neurosurgeons. Preoperative intracranial pressure was assessed through lumbar puncture. A standard VPS procedure (usually right side) was performed: the patient in a supine position, head turned to the left, and right shoulder elevated. Routine disinfection and draping were performed on the head, chest, and abdomen. A curved scalp incision, 2.5 cm lateral to the midline within the right hairline, was made. The ventricle catheter was inserted into the anterior horn of the lateral ventricle via a burr hole at Kocher’s point, typically on the right side to minimize the risk of injury to the dominant hemisphere. A 5 cm subxiphoid abdominal incision was created for the placement of the peritoneal catheter. In cases where patients presented with abdominal adhesions, laparoscopic guidance was employed for the placement of the peritoneal catheter. The initial pressure setting of the shunt pump was documented and subsequently adjusted based on clinical symptoms and imaging findings. The observation period after pressure adjustment generally spanned 1 to 2 weeks.

The adjustable pressure shunt valve (Medtronic, USA) has five pressure levels: 0.5 (15–25 mmH_2_O, 1 mmH_2_O = 0.0098 kPa), 1.0 (35–55 mmH_2_O), 1.5 (70–90 mmH_2_O), 2.0 (105–125 mmH_2_O), and 2.5 (135–155 mmH_2_O).

### 2.5 Data collection

The data collected encompassed several variables: gender, age, history of hypertension and diabetes, history of craniotomy and cranial defect, preoperative pneumonia and Glasgow Coma Scale (GCS). Additionally, primary intracranial pathologies were recorded, including normal pressure hydrocephalus, trauma, spontaneous intracranial hemorrhage, tumor, and inflammation. Preoperative lumbar puncture pressure was measured, and hydrocephalus type was categorized into three types: low pressure hydrocephalus (LPH, CSF pressure < 80 mmH_2_O), normal pressure hydrocephalus (NPH, 80–180 mmH_2_O), and high pressure hydrocephalus (HPH, CSF pressure > 180mmH_2_O). Laboratory values obtained within 5 days preoperatively included the international normalized ratio (INR), prothrombin time (PT), activated partial thromboplastin time (APTT), serum white blood cell count, platelet count, neutrophil count, and lymphocyte count. Postoperative laboratory values for neutrophil count, lymphocyte count, and platelet count were obtained on the morning of day 1. The presence of peri-catheter edema on the first postoperative CT scan was also noted.

For patients diagnosed with DICH, additional data were collected, including the date of hemorrhage onset, hemorrhage location, presence of symptoms, GOS, and all cranial CT scans obtained before and after VPS placement. Hematoma volume was calculated using the 3D Slicer software (version 5.8.1).

### 2.6 Statistical analysis

Data analysis was conducted utilizing SPSS version 26.0. The distribution of continuous variables was assessed using the Kolmogorov–Smirnov test. For continuous data that did not follow a normal distribution, such as age, blood pressure, bleeding time, and bleeding volume, the Mann–Whitney U test was employed, with results reported as the median (50%) accompanied by the interquartile range (IQR). Categorical variables are presented as frequencies and percentages. The Mann–Whitney test was used for ordered categorical variables, while unordered categorical variables were analyzed using Pearson’s chi-square test, the continuity-corrected chi-square test, or Fisher’s exact test. Factors with *p* < 0.05 identified in the univariate analysis were incorporated into the multivariate logistic regression model for evaluation, and the independent influencing factors were obtained. R software (Version 4.4.3) was used to construct the nomogram predictive model, and internal validation was performed by plotting the receiver operating characteristic (ROC) curve and calibration curve. A *p*-value < 0.05 was considered statistically significant.

## 3 Results

### 3.1 Clinical characteristics of patients with DICH after VPS

Among the cohort of 266 hydrocephalus patients underwent VPS procedures, 46 individuals (17.3%) developed DICH. The mean time to onset of DICH was 5.7 days postoperatively, with a standard deviation of 2.62 days, and a range of 2–13 days. Symptomatic manifestations, including vomiting, seizures, and decreased consciousness, were observed in 10 patients, whereas 36 patients remained asymptomatic. The median hemorrhagic volume was 0.85 mL, with an interquartile range of 0.28–3.23 mL, and the volumes spanned from 0.13 to 75.47 mL. Hemorrhage volumes were classified into three categories: less than 1 mL (25 cases), between 1 and 15 mL (17 cases), and greater than 15 mL (4 cases). The anatomical locations of hemorrhages were identified as follows: catheter tract only (17 cases), ventricular only (19 cases), combined catheter tract and ventricular (7 cases), and catheter tract with other locations (3 cases). Eight patients underwent surgical intervention, and 38 patients received conservative management. At discharge, 8 patients had a favorable outcome (GOS > 3), whereas 38 patients exhibited an unfavorable outcome (GOS ≤ 3).

### 3.2 Univariate analysis of DICH after VPS in hydrocephalus patients

This study included 266 patients, among whom 220 individuals (82.7%) did not develop DICH and were categorized as Group A, while 46 individuals (17.3%) developed DICH and were categorized as Group B. Significant differences (*p* < 0.05) were found between the two groups with respect to age, history of hypertension, history of craniotomy, preoperative NLR, postoperative NLR, and NLRR. No significant differences (*p* > 0.05) were observed between the groups concerning the preoperative GCS, preoperative PT, preoperative APTT, preoperative INR, preoperative PLT, postoperative PLR, history of diabetes, history of cranial defect, preoperative pneumonia, primary intracranial pathology, type of hydrocephalus, number of catheter passes, catheter placement side, initial shunt pressure setting, peri-catheter edema, or shunt pressure adjustment (Table 1).

**Table 1:** Univariate analysis of delayed intracranial hemorrhage following ventriculoperitoneal shunt in hydrocephalus patients.

Variables	A Group (*n* = 220)	B Group (*n* = 46)	*χ*^2^/Z	*P*
Demographics				
Male sex, *n*(%)	120(54.5)	32(69.6)	3.505	0.061
Age (y), median [IQR]	60.00 (53.00, 68.00)	65.00 (59.25, 73.00)	3.247	0.001*
Clinical history, *n*(%)				
Hypertension	89 (40.5)	26 (56.5)	4.002	0.045*
Diabetes mellitus	28 (12.7)	9 (19.6)	1.486	0.223
Preoperative pneumonia	74 (33.6)	17 (37.0)	0.189	0.666
Craniotomy	111 (50.5)	32 (69.6)	5.589	0.018*
History of skull defect, *n*(%)			0.362	0.547
No defect	134 (60.9)	21 (45.7)		
With defect	62(28.2)	15(32.6)		
Repaired before shunt surgery	24(10.9)	10(21.7)		
Preoperative GCS, median [IQR]	12.00 (9.00, 15.00)	11.50 (9.00, 15.00)	0.060	0.952
Puncture site, *n*(%)			1.043	0.307
Left precornu	69 (31.4)	18 (39.1)		
Right precornu	151 (68.6)	28 (60.9)		
Number of punctures, *n*(%)			0.415	0.678
1 time	196 (89.1)	40 (87.0)		
2 times	20 (9.1)	5 (10.9)		
3 times	4 (1.8)	1 (2.2)		
Primary intracranial lesion, *n*(%)			6.742	0.125
Normal hydrocephalus	16 (7.3)	1 (2.2)		
Trauma	89 (40.5)	26 (56.5)		
ICH	86 (39.1)	17 (37.0)		
Tumor	24 (11.0)	1 (2.2)		
Inflammation	5 (2.3)	1 (2.2)		
Hydrocephalus type, *n*(%)			−0.651	0.515
LPH	22 (10.0)	4 (8.7)		
NPH	160 (72.7)	37 (80.4)		
HPH	38 (17.3)	5 (10.9)		
Laboratory test, median [IQR]				
Pre-PT (s)	11.80 (11.20, 12.50)	11.70 (11.10, 12.38)	−0.498	0.619
Pre-APTT (s)	29.95 (28.20, 32.15)	30.95 (29.07, 32.58)	1.426	0.154
Pre-INR (ratio)	1.04 (0.99, 1.10)	1.04 (0.96, 1.09)	−0.519	0.604
Pre-PLT (*10^9^/μL)	236.50 (183.00, 285.25)	210.50 (178.50, 261.25)	−1.525	0.127
Pre-NLR (ratio)	3.25 (1.96, 5.00)	2.34 (1.53, 3.07)	−2.986	0.003*
Post-PLR (ratio)	180.65 (138.09, 252.68)	191.92 (129.67, 271.31)	0.110	0.913
Post-NLR (ratio)	5.73 (3.83, 8.11)	6.40 (5.45, 10.45)	2.601	0.009*
NLRR (ratio)	1.80 (1.27, 2.84)	3.50 (2.14, 4.40)	5.202	<0.001*
Surgical related indicators, *n*(%)				
Initial pressure of vale system			−0.919	0.358
1.0	35 (16.0)	8 (17.4)		
1.5	107 (48.6)	25 (54.4)		
2.0	71 (32.3)	13 (28.3)		
2.5	7 (3.2)	0 (0.00)		
Manipulation of valve system	70 (31.8)	17 (37.0)	0.456	0.499
Brain edema around catheter	60 (27.3)	15 (32.6)	0.535	0.464

GCS, Glasgow Coma Scale; ICH, spontaneous intracerebral hemorrhage; LPH, low pressure hydrocephalus; NPH, normal pressure hydrocephalus; HPH, high pressure hydrocephalus; Pre-PT, preoperative prothrombin time; Pre-APTT, preoperative activated partial thromboplastin time; Pre-INR, preoperative international normalized ration; Pre-PLT, preoperative serum thrombocyte; Pre-NLR, preoperative neutrophil-to-lymphocyte ratio; Post-PLR, postoperative platelet-to-lymphocyte ratio; Post-NLR, postoperative neutrophil-to-lymphocyte ratio; NLRR, a ratio of post-NLR to pre-NLR. *P < 0.05.

### 3.3 Multivariate logistic regression analysis of DICH after VPS in hydrocephalus patients

In a binary multivariate logistic regression analysis, variables such as age, history of hypertension, history of craniotomy, preoperative NLR, postoperative NLR, and NLRR were selected as independent variables, with the occurrence of DICH serving as the dependent variable. The analysis revealed that age (OR = 1.061), history of craniotomy (OR = 2.676), and NLRR (OR = 1.931) were significant independent predictors for DICH following VPS (*p* < 0.05) (Table 2).

**Table 2:** Multivariate logistic regression analysis of delayed intracranial hemorrhage following ventriculoperitoneal shunt in hydrocephalus patients.

Variables	B	*p* value	Odd ratios	95%CI
Age	0.059	0.003*	1.061	1.021–1.103
Hypertension	0.414	0.283	1.512	0.711–3.217
Craniotomy	0.984	0.017*	2.676	1.196–5.989
Pre-NLR	0.079	0.399	1.083	0.900–1.302
Post-NLR	−0.006	0.909	0.994	0.899–1.099
NLRR	0.658	<0.001*	1.931	1.373–2.717

95%CI, confidence interval; Pre-NLR, preoperative neutrophil-to-lymphocyte ratio; Post-NLR, postoperative neutrophil-to-lymphocyte ratio; NLRR, a ratio of post-NLR to pre-NLR. *P<0.05.

### 3.4 Construction of the nomogram model for delayed intracranial hemorrhage after ventriculoperitoneal shunt in patients with hydrocephalus

**Figure 2:**
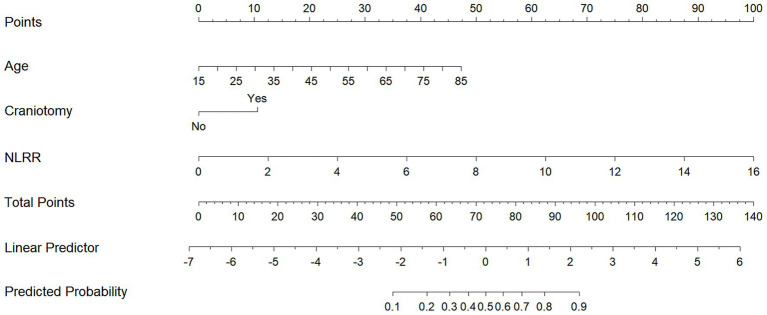
Nomogram model construction for predicting delayed intracranial hemorrhage after ventriculoperitoneal shunt in hydrocephalus patients.

The nomogram predictive model for DICH following VPS in hydrocephalus patients was constructed based on the influencing factors (age, craniotomy, and NLRR) identified by multivariate logistic regression analysis (Figure 2). For example, a 65-year-old patient without history of craniotomy and an NLRR of 6. The scores for each factor were 34 points, 0 points, and 37 points respectively, with a total score of 71 points. According to the nomogram predictive model, the probability of DICH occurring in this hydrocephalus patient after VPS surgery was 47.5%.

### 3.5 The predictive efficacy of delayed intracranial hemorrhage after ventriculoperitoneal shunt in patients with hydrocephalus

**Figure 3:**
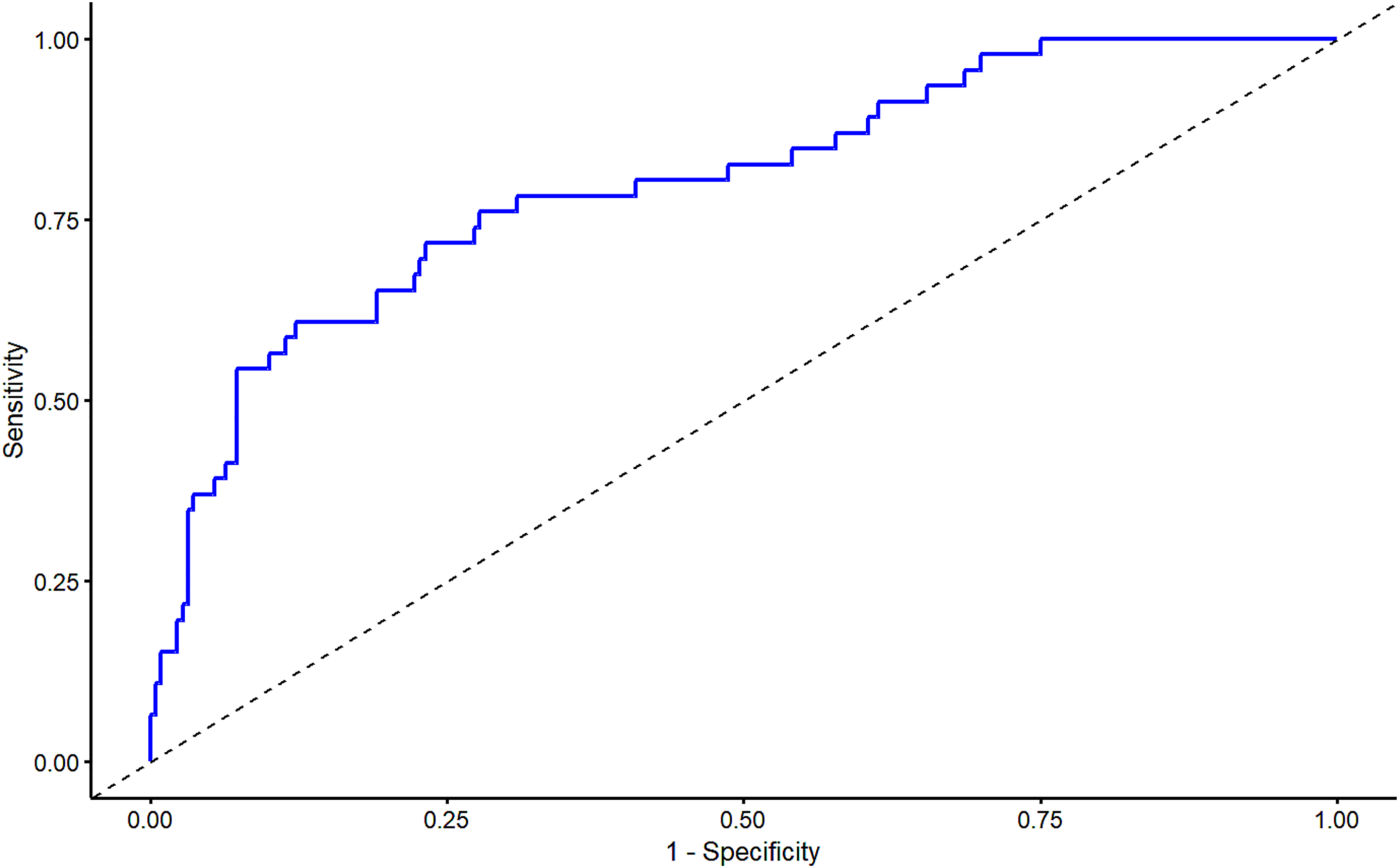
Receiver operating characteristic curve of the nomogram model for predicting delayed intracranial hemorrhage after ventriculoperitoneal shunt in hydrocephalus patients.

**Figure 4:**
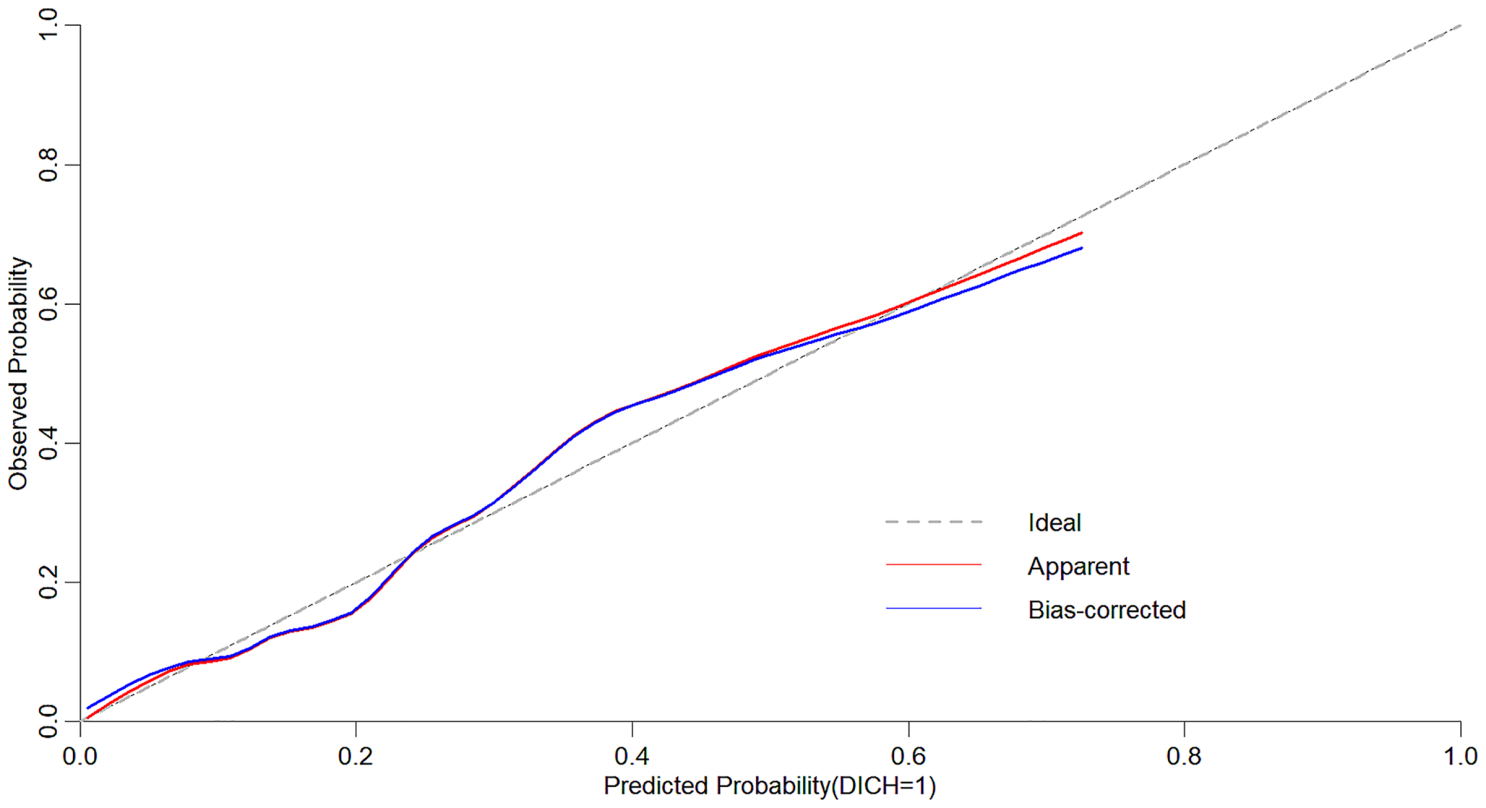
Calibration curve of the nomogram model for predicting delayed intracranial hemorrhage after ventriculoperitoneal shunt in hydrocephalus patients.

The results of ROC curve analysis showed that the Area Under the Curve (AUC) of the Logistic model for predicting the probability of DICH after VPS in patients with hydrocephalus was 0.80 (95% CI: 0.73–0.87). The sensitivity was 87.7%, the specificity was 60.9%, the positive predictive value (PPV) was 91.5%, the negative predictive value (NPV) was 50.9%, and the cutoff value was 0.254 (Figure 3). The slope of the calibration curve of the nomogram model for predicting DICH after VPS in hydrocephalus patients was close to 1. Results of the Hosmer-Lemeshow test showed that X^2^ = 10.145 and *p* = 0.255, which indicated a high degree of accuracy of the mode (Figure 4).

## 4 Discussion

Hydrocephalus, characterized by abnormal production or impaired absorption of cerebrospinal fluid leading to ventricular or subarachnoid space enlargement, presents with a range of symptoms including headache, vomiting, blurred vision, papilledema, and occasionally diplopia, vertigo, and seizures. In severe cases, it may lead to impaired consciousness and incontinence (9). DICH was first described by Matsumura et al. in 1985 (10), and is considered as a serious complication that can adversely affect patient outcomes (11). DICH is defined as new hemorrhage occurring along the shunt catheter path or within the ventricles, as detected on follow-up imaging 5 to 10 days postoperatively (5), which is absent on the initial CT scans obtained within 24 h after VPS placement, thereby excluding early postoperative hemorrhage or anticoagulation-related bleeding. The high-risk period for DICH following VPS typically occurs within the first week (12).

Notably, the results of a retrospective study indicated that the probability of developing DICH after VPS was only 3.8% (5). Whereas, a study conducted in South Korean documented a substantially higher incidence of 23.7% (13). Our study identified an incidence rate of 17.3%, which is intermediate between above two findings, while the incidence of symptomatic DICH was only 3.76%, aligning with the findings of Hou et al. (14). These discrepancies may be due to differences in sample size, inclusion and exclusion criteria (12), and our inclusion of asymptomatic occult hemorrhages smaller than 1 mL.

The mechanisms of DICH remain incompletely elucidated; however, potential contributing factors may include: 1. progressive vascular erosion and inflammatory responses elicited by the catheter as a foreign body (15); 2. increased fragility of brain tissue associated with aging, underlying primary brain disease, or a history of craniotomy (13); 3. reduced tamponade effect resulting from decreased intracranial pressure following CSF drainage and shunt pressure adjustments (16, 17); 4. coagulation disorders or the use of anticoagulant or antiplatelet medications (8, 18, 19). Our univariate analysis identified the significant associations between DICH and those factors such as age, hypertension, a history of craniotomy, preoperative NLR, postoperative NLR, and NLRR (*p* < 0.05). Multivariate analysis further confirmed advanced age, a history of craniotomy, and NLRR as independent risk factors for DICH (*p* < 0.05). Overall, the etiology and mechanisms of DICH are likely multifactorial and interrelated, rather than attributable to a singular causative factor.

This study confirmed advanced age as an independent risk factor for DICH (OR = 1.061 per year, 95% CI: 1.021–1.103), consistent with previous findings (4, 13). The OR value implies that each additional year of age elevates the odds of DICH by 6.1%, which is biologically plausible due to age-related vascular and parenchymal changes: (1) Cerebral atherosclerosis and vascular amyloidosis, common in older adults, reduce the structural integrity of cerebral microvasculature, making vessels more susceptible to mechanical injury from shunt catheter insertion or postoperative hemodynamic fluctuations; (2) Age-related decline in cerebral compliance and impaired autoregulation may exacerbate vascular shear stress after CSF drainage, further increasing hemorrhage risk. Clinically, this translates to a notable risk increment in elderly populations.

Furthermore, the observed correlation between prior craniotomy and DICH aligns with earlier studies (13, 18). This association may be related to increased fragility of brain tissue and reduced compliance following craniotomy, or the formation of adhesions between the arachnoid membrane and cerebral small vessels, which predispose to hemorrhage during shunt catheter insertion. While some studies have identified peri-catheter edema on initial postoperative CT scans as an independent risk factor for DICH, potentially related to impaired cortical venous drainage or coagulation abnormalities caused by catheter insertion (5, 6). However, this significant association was not found in our study. The discrepancy may be attributed to meticulous intraoperative care aimed at avoiding damage to the draining veins at the puncture site.

Elevated NLRR was identified as an independent risk factor for DICH in the study, consistent with the findings of Li et al. (20), and suggesting that inflammation plays a significant role in the development of DICH. Some studies have demonstrated that decreased neutrophils and increased lymphocytes may reduce blood–brain barrier disruption (21–23). Thus, an elevated NLRR, which reflects either an increase in neutrophils or a decrease lymphocytes, could potentially exacerbate the degradation of blood–brain barrier and contribute to the onset induce DICH. As a novel dynamic inflammatory marker, NLRR provides insight into the perioperative inflammatory status, and may assist in identifying patients who require intensified postoperative management to prevent DICH.

The nomogram enables the intuitive visualization of predictive variables derived from multivariate logistic regression analysis; by calculating the total predictive score, it helps clinicians understand the probability of a specific adverse event occurring (24). Currently, there is a paucity of studies that have constructed nomogram models to identify factors influencing the development of DICH following VPS surgery. Based on three influencing factors—age, history of craniotomy, and NLRR—this study developed a nomogram model for predicting the risk of DICH after VPS surgery in patients with hydrocephalus. The model was validated using the ROC curve and calibration curve. Results showed that the AUC was 0.80, with the slope of the calibration curve approaching 1, indicating that the model exhibits good discriminative ability and accuracy. In clinical practice, this model allows for individualized prediction of DICH risk in hydrocephalus patients after VPS surgery based on each risk factor, facilitating the timely identification of high-risk patients and the implementation of scientific preventive and control measures in advance.

In conclusion, advanced age, a history of craniotomy, and elevated NLRR are independent risk factors for DICH following VPS in hydrocephalus patients. Therefore, for patients presenting with any of these risk factors, increasing the frequency of postoperative cranial CT scans may enhance the sensitivity for detecting small, asymptomatic hemorrhages. This could facilitate early intervention, thereby minimizing serious complications and improving patients’ prognosis. However, this study has several limitations: (1) the conclusions are derived from a retrospective analysis with a relatively small sample size, which may introduce potential bias and confounding variables; (2) The study considered a limited number of influencing factors. For instance, due to the exclusion criteria, we did not analyze the history of antiplatelet/anticoagulant therapy. Additionally, we did not record detailed grading of hypertension severity (e.g., mild, moderate, or severe based on blood pressure levels or duration), nor did we evaluate operator-specific factors (such as surgeons’ years of experience, individual technical preferences, or procedural variations), all of which may potentially impact the occurrence of DICH; (3) the data were collected from a single center, lacking external validation. Future work should focus on expanding the sample size and incorporating multi-center data to achieve external validation.

## Data Availability

The original contributions presented in the study are included in the article/supplementary material, further inquiries can be directed to the corresponding author.
